# Fetal Thigh Circumference Nomograms Across Gestational Ages: A Retrospective Study

**DOI:** 10.3390/jpm15070265

**Published:** 2025-06-22

**Authors:** Ferdinando Antonio Gulino, Giorgio Arcarese, Giosuè Giordano Incognito, Giuliana Orlandi, Olimpia Gabrielli, Antonia Lettieri, Luigi Manzo, Laura Letizia Mazzarelli, Giordana Sica, Letizia Di Meglio, Lavinia Di Meglio, Attilio Tuscano, Sara Occhipinti, Maurizio Guida, Aniello Di Meglio

**Affiliations:** 1 Unit of Gynecology and Obstetrics, Department of Human Pathology of Adults and Developmental Age, “G. Martino” University Hospital, 98122 Messina, Italy; docferdi@hotmail.it (F.A.G.); giorgio.arcarese@libero.it (G.A.); 2 Department of General Surgery and Medical Surgical Specialties, University of Catania, 95123 Catania, Italy; saraocchipinti91@gmail.com; 3 Department of Neuroscience, Reproductive Sciences and Dentistry, School of Medicine, University of Naples Federico II, 80131 Naples, Italy; giulianaorlandi@msn.com (G.O.); olimpia.gabrielli3@gmail.com (O.G.); luigimanzo93@libero.it (L.M.); lauramazzarelli@gmail.com (L.L.M.); mauguida@unina.it (M.G.); 4 Diagnostica Ecografica e Prenatale di A. Di Meglio, 80133 Naples, Italy; antonia_lettieri@libero.it (A.L.); giordanasica@icloud.com (G.S.); aniellodimeglio@gmail.com (A.D.M.); 5 Radiology Department, School of Medicine, University of Milan, 20133 Milan, Italy; letiziadimeglio@gmail.com; 6 Pediatric Department, Bambino Gesù Children’s Research Hospital, IRCCS, 00165 Rome, Italy; laviniadimeglio@gmail.com; 7 Department of Obstetrics and Gynaecology, “Bianchi-Melacrino-Morelli” Hospital, 89128 Reggio Calabria, Italy; attiliotuscano@gmail.com

**Keywords:** biometry, gestational age, ultrasound, prenatal diagnosis

## Abstract

**Background/Objectives**: Fetal thigh circumference (ThC) may be a valuable parameter for assessing fetal growth. Thus, this study aimed to establish reference ranges for ThC across gestational ages (GA). **Methods**: This retrospective study included singleton pregnancies between 12 and 38 weeks of gestation. ThC measurements were obtained during routine ultrasound examinations. GA was confirmed through the last menstrual period and first-trimester crown–rump length measurements. Percentile ranges for ThC were calculated for each gestational week, and statistical analyses evaluated the relationship between ThC and GA. **Results**: 48,841 singleton pregnancies were included. A positive correlation was observed between ThC and GA, with ThC values increasing progressively from 12 to 38 weeks. The study established the 10th, 50th, and 90th percentile ranges for ThC, providing reference values for clinical assessments. **Conclusions**: This study provides reference ranges for fetal ThC across a wide GA range, highlighting its potential as a tool in prenatal care. ThC may offer an additional parameter for monitoring fetal growth, especially when standard measurements are challenging. Further research should investigate the integration of ThC with other fetal growth parameters to enhance its clinical utility. Additionally, these nomograms can be used to assess their usefulness in certain conditions, such as intrauterine growth restriction (IUGR), macrosomia, and congenital skeletal dysplasias.

## 1. Introduction

Accurate fetal growth assessment is a cornerstone of prenatal care, offering essential insights into fetal health and development. Standard biometric parameters, such as head circumference, biparietal diameter, abdominal circumference, and femur length (FL), are routinely used to monitor fetal growth and identify potential abnormalities [1]. These measurements form the basis of obstetric practice, providing clinicians with reliable tools to evaluate whether a fetus is growing within expected norms or deviating in a manner that may require intervention. However, increasing interest in additional parameters may provide a more comprehensive picture of fetal growth, especially in cases where traditional measurements are challenging to obtain or may not fully capture certain growth dynamics [2]. Fetal thigh circumference (ThC) may emerge as a valuable parameter in this context, reflecting both skeletal and soft tissue development and offering insights into fetal growth and musculoskeletal health.

The thigh comprises complex skeletal, muscular, vascular, and connective tissues that play key roles in fetal growth. From an embryological perspective, its development originates from mesodermal structures, which subsequently differentiate into the bones and muscles of the lower limbs. ThC captures bone and soft tissue growth, providing a unique window into these developmental processes [3].

Some evidence has demonstrated that ThC correlates well with gestational age (GA) and birth weight, suggesting that it could be an effective adjunct to other growth assessment tools, particularly in high-risk pregnancies [4,5]. Additionally, this measure may be particularly valuable in detecting intrauterine growth restriction (IUGR), a pregnancy complication associated with increased morbidity and mortality. IUGR is often characterized by reduced fetal muscle mass and soft tissue, both of which can influence ThC. By including ThC as part of routine biometric assessments, clinicians could improve the early detection of IUGR, potentially allowing for timely interventions that mitigate associated risks. It may also aid in the detection of macrosomia, which is linked to increased risks during labor and delivery, as well as metabolic complications in neonates. Traditional parameters, such as abdominal circumference, are widely used to estimate macrosomia, but incorporating ThC could enhance predictive accuracy by providing a more detailed view of fetal growth patterns. Studies have shown that including soft tissue parameters, like ThC, improves the ability to predict macrosomia in the third trimester, thereby allowing for better management of pregnancies at risk [6,7]. Such advancements underscore the broader clinical implications of integrating ThC measurements into standard prenatal protocols, particularly in pregnancies complicated by metabolic conditions such as gestational diabetes. Moreover, ThC may be particularly useful in cases where the fetal head is engaged in the pelvis, making it difficult to obtain head circumference measurements. ThC can serve as an alternative marker for estimating fetal weight and overall size, offering flexibility in prenatal assessments [8,9]. This adaptability makes ThC a highly practical tool in diverse clinical scenarios, including those involving late-pregnancy evaluations where traditional metrics are more challenging to obtain.

Despite its clinical potential, reference ranges for ThC across GAs are not as well established as those for other biometric parameters. Thus, this study aimed to establish comprehensive reference ranges for ThC across gestation, based on a large cohort of pregnancies. By analyzing the correlation between ThC and GA, the study aimed to provide normative data that could enhance fetal growth assessments and complement existing biometric measurements. In doing so, it seeks to fill a critical gap in obstetric practice, paving the way for more comprehensive and individualized approaches to prenatal care.

## 2. Materials and Methods

### 2.1. Inclusion Criteria

This is a retrospective study that includes only singleton pregnancies without fetal anomalies or significant growth disturbances, such as IUGR or macrosomia. Specifically, for pregnancies beyond the first trimester, fetuses with estimated weights or abdominal circumference between the 10th and 90th percentiles for gestational age were selected [10]. For pregnancies in the first trimester, inclusion was based on crown–rump length (CRL) measurements falling within the range expected for GA. Participants had negative histories of systemic diseases.

### 2.2. Ultrasound Examinations

ThC measurements were taken during routine US examinations in the first, second, and third trimesters by a single experienced sonographer. Each fetus underwent a single measurement of ThC at the respective gestational age. GA fractions were rounded to the nearest whole week, with ≤4 days assigned to the current week and >5 days to the subsequent week. US examinations were conducted using Aloka (Aloka Co., Ltd., Tokyo, Japan) and Voluson E10 (GE Healthcare Ultrasound, Milwaukee, WI, USA) machines. Both devices were equipped with curved linear array transabdominal transducers (2–5 MHz) and transvaginal probes (4–8 MHz). ThC was measured in the transverse plane, perpendicular to the longitudinal axis of the femur. The imaging plane was carefully adjusted to obtain a circular cross-section of the fetal thigh, ensuring the most significant possible cross-sectional area while maintaining the femur centrally positioned within the scan field. The reference point for the ThC measurement was standardized at the junction between the upper and middle thirds of the fetal thigh, corresponding to the level of the proximal nutrient foramen of the femur. The cross-sectional area was assessed at the point where the soft tissue and musculature surrounding the femur reached its maximum thickness. A transvaginal approach was utilized when transabdominal imaging did not permit optimal visualization.

### 2.3. Statistical Analysis

Statistical analyses were performed using GraphPad Prism (version 8.4.2 for Windows, GraphPad Software, San Diego, CA, USA) and IBM^®^ SPSS^®^ (version 21.0, SPSS Inc., Chicago, IL, USA). Descriptive statistics, including the mean and standard deviation, were calculated for participant characteristics. The normality of data distribution was assessed using the Kolmogorov–Smirnov test. Spearman’s rank correlation coefficient was used to examine the relationship between GA and ThC, with scatter plots illustrating data trends. Linear regression analysis was applied to model the association between GA and ThC. A *p*-value of less than 0.05 was considered statistically significant.

## 3. Results

A total of 48,841 US examinations were performed, allowing for the measurement of ThC in fetuses from 12 to 38 weeks of gestation. ThC measurements were collected for all participants within this GA range, and the values are summarized in Table 1. The data reflect the 10th, 50th, and 90th percentiles of ThC, expressed in centimeters (cm), for each GA, providing a distribution of measurements across the study population.

**Figure 1:**
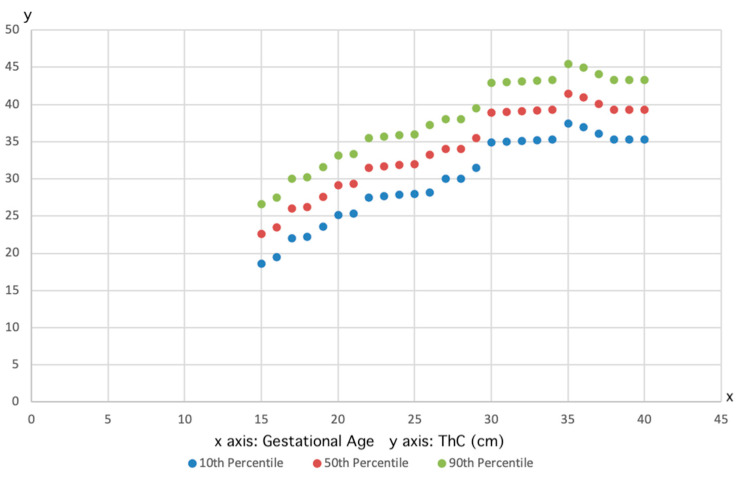
Plot showing the fetal thigh circumference measurements and the fitted 10th (lower line), 50th (median line), and 90th (higher line) percentiles for gestational age.

In addition, a consistent increase in ThC with advancing GA was observed across the entire study cohort. This positive correlation between GA and ThC is supported by the plot of observed measurements, which align with the fitted 10th, 50th, and 90th percentiles for each week of gestation, as shown in Figure 1.

No significant changes in the sonolucency of the thigh section were noted across the range of GAs. The thigh section remained consistent in appearance, with a circular cross-section and a centrally located hyperechogenic femur, observable across all GA measurements.

## 4. Discussion

### 4.1. Summary and Interpretation of Results

This study provides comprehensive reference ranges for ThC as a function of GA based on data from a large cohort of pregnancies. A clear positive correlation between GA and ThC was observed, with an increase in ThC corresponding to fetal growth stages, which may reflect changes in musculoskeletal development. These results establish normative curves that can assist clinicians in monitoring fetal growth and detecting deviations from expected patterns, which may prompt further evaluation for conditions such as IUGR or macrosomia in a clinical context. The steady progression of ThC values over time also supports its use in monitoring normal fetal development.

### 4.2. Insights from the Existing Literature

The role of ThC in growth assessment has been explored in a limited number of studies, but existing evidence highlights its potential clinical utility.

Maruotti et al. [11] conducted a meta-analysis that showed fetal thigh and abdominal measurements as reliable predictors of macrosomia, with a pooled sensitivity of 80% and specificity of 95%. The authors also suggested that ThC, when used in conjunction with abdominal measurement, can enhance the accuracy of macrosomia predictions in late pregnancy. Such findings reinforce the potential utility of ThC as a critical parameter for evaluating fetal growth patterns beyond the typical biometric measurements.

ThC also may play a role in assessing soft tissue development, as discussed in two studies by Lee and colleagues [4,5], who introduced the fractional thigh volume (TVol) as an additional parameter for assessing soft tissue and growth dynamics. TVol includes 50% of the femoral diaphysis length, allowing for a comprehensive evaluation of thigh soft tissue. The present study’s ThC findings complement their results, as they underscore the importance of detailed ThC measurements in understanding fetal body composition and growth trajectory.

Although fetuses with congenital anomalies were excluded from our study cohort, previous research has discussed the relevance of ThC in the context of congenital anomalies. For example, skeletal dysplasias, such as thanatophoric dysplasia (TD) and achondroplasia, are often characterized by limb shortening and abnormal bone structure, which could manifest as reduced ThC. In TD, particularly in type I, femoral curvature (“telephone receiver” femurs) and extreme rhizomelia are distinguishing features, while type II presents with straight but shortened femurs [6]. In cases of achondroplasia, ThC may also be reduced due to overall limb shortening and curvature, supporting the utility of ThC as an indirect marker of certain dysplasias. Furthermore, conditions such as osteogenesis imperfecta, which involves skeletal demineralization, can impact ThC due to brittle bone structure that affects limb development and, ultimately, ThC [12]. This points to ThC’s potential utility in prenatal screening for congenital skeletal disorders. In addition to skeletal dysplasias, ThC can be affected by conditions like Klippel–Trenaunay–Weber syndrome, which may present prenatally as congenital lymphangiohemangioma. In this condition, abnormal masses can distort normal thigh anatomy, leading to false positive ThC measurements due to space-occupying lesions in the thigh region. As described in case reports, massive cystic areas in the lower limbs of affected fetuses can cause ThC to deviate significantly from normative values, highlighting the importance of comprehensive anatomical assessments in conjunction with ThC measurements [13].

Further, as discussed by Kandil et al. [8], ThC combined with FL measurements may be useful for estimating fetal weight, particularly when head measurements are not feasible due to engagement or other positional factors. Therefore, ThC may offer a viable alternative or complement to traditional biometry, especially in difficult measurement situations. Kandil’s study, which employs Isobe’s formula to combine FL and ThC, demonstrated that these parameters can yield accurate fetal weight estimations, particularly in late pregnancy.

### 4.3. Implications for Clinical Practice

This study emphasizes the potential of ThC as an accessible and reliable parameter for evaluating fetal growth. By incorporating ThC measurements into routine US assessments, clinicians can improve the detection of growth abnormalities, particularly in populations at risk for IUGR and macrosomia. By offering a comprehensive perspective on both skeletal and soft tissue development, ThC complements traditional biometry and enhances the ability to monitor fetal well-being. ThC can aid in identifying certain skeletal dysplasias, complementing other measurements such as FL. Given its consistency with gestational progression, ThC can serve as a longitudinal growth marker, tracking normal fetal development. This can facilitate more precise decision making, allowing for earlier interventions and tailored management strategies if deviations are detected. For example, pregnancies identified as high risk for IUGR could benefit from closer monitoring and timely delivery planning, potentially reducing perinatal morbidity and mortality. Similarly, accurate detection of macrosomia could inform decisions regarding the mode of delivery, mitigating risks associated with shoulder dystocia or cesarean delivery. ThC measurements can be especially valuable in cases where traditional biometry parameters are difficult to obtain. This may be particularly relevant in pregnancies where fetal positioning complicates head or abdominal circumference measurements. ThC provides an additional option for estimating fetal weight, broadening the scope of fetal assessments and offering clinicians a more flexible approach to growth monitoring.

### 4.4. Strengths and Limitations

The study’s large sample size allows for the development of robust percentile ranges that can be generalized across a wide population. The data cover a broad range of GAs, offering insight into fetal thigh growth throughout significant developmental stages. The use of standardized US equipment and measurement protocols contributes to the accuracy and reproducibility of the findings.

However, some limitations should be acknowledged. First, the study cohort was restricted to fetuses with estimated weights within the 10th to 90th percentiles, which excludes extreme growth patterns. While this approach was necessary to establish normative data, it may limit the application of these findings in cases of severe IUGR or macrosomia. Additionally, the study did not account for potential influences from maternal factors, such as gestational diabetes or obesity, which can affect fetal growth and ThC measurements. Including such variables could provide further insight into the relationship between maternal health and ThC. Moreover, the study does not address potential variations related to maternal factors, such as ethnicity, nutritional status, or genetic predispositions. This limitation is mainly due to the study’s retrospective nature, which did not allow consistent and reliable retrieval of detailed maternal information. We recognize this as a potential constraint on the generalizability of the findings and suggest that future prospective studies include maternal variables to contextualize ThC measurements better and enhance their clinical utility. These factors could influence fetal growth patterns and may warrant further investigation to refine the applicability of ThC measurements in diverse populations. Moreover, future prospective studies could provide additional insights into the trajectory of ThC growth and its relationship with perinatal outcomes. Lastly, while the study provides percentile data for ThC, it does not explore other lower limb parameters that may complement ThC in assessing fetal growth. Future studies might benefit from examining the combined use of ThC with other measurements to develop a more holistic approach to fetal growth monitoring.

## 5. Conclusions

This study establishes reference ranges for ThC across a wide range of GAs, providing clinicians with a valuable tool for monitoring fetal growth. The results emphasize ThC’s utility as an additional biometric parameter that correlates closely with gestational progression. The development of percentile ranges based on a large, diverse sample enhances the applicability of ThC in clinical settings, allowing for more precise growth assessments across different stages of pregnancy. Future research should explore the integration of ThC with other growth parameters, such as fractional thigh volume and femur length, to create a more holistic approach to fetal growth monitoring. Additionally, further studies examining the influence of maternal factors on ThC could deepen the understanding of how maternal health impacts fetal development. Expanding the scope of fetal assessments through such integrated measurements has the potential to improve prenatal care outcomes, enabling early and accurate detection of fetal growth abnormalities and facilitating timely, targeted interventions.

## Figures and Tables

**Table 1:** Predicted 10th, 50th, and 90th percentiles of the fetal thigh circumference (cm) by gestational age (weeks).

Number of Cases	Gestational Age (Weeks)	10th Percentile (cm)	50th Percentile (cm)	90th Percentile (cm)
2600	12	4.6	6.8	9
1250	13	4.7	6.9	9.1
365	15	7.9	10.1	12.3
720	16	11.3	13.5	15.7
770	17	11.9	14.1	16.3
1150	18	12.9	15.1	17.3
8060	19	15.1	17.3	19.5
14,060	20	16.8	19	21.2
7630	21	17.7	19.9	22.1
2340	22	19.8	22	24.2
910	23	20.2	22.4	24.6
560	24	21.8	24	26.2
570	25	23.5	25.7	27.9
610	26	26.5	28.7	30.9
540	27	26.6	28.8	31
550	28	26.7	28.9	31.1
670	29	28.8	31	33.2
920	30	31.6	33.8	36
1030	31	31.6	33.8	36
966	32	31.7	33.9	36.1
820	33	31.4	33.6	35.8
650	34	32.9	35.1	37.3
540	35	34	36.2	38.4
420	36	36.3	38.5	40.7
140	38	36.3	38.5	40.7

## Data Availability

Data are available upon reasonable request.
